# Defective bone repair in mast cell-deficient *Cpa3^Cre/+^* mice

**DOI:** 10.1371/journal.pone.0174396

**Published:** 2017-03-28

**Authors:** Jose Luis Ramirez-GarciaLuna, Daniel Chan, Robert Samberg, Mira Abou-Rjeili, Timothy H. Wong, Ailian Li, Thorsten B. Feyerabend, Hans-Reimer Rodewald, Janet E. Henderson, Paul A. Martineau

**Affiliations:** 1 Bone Engineering Labs, Research Institute-McGill University Health Centre. Montreal General Hospital C10.160, Cedar Ave., Montreal, QC, Canada; 2 Experimental Surgery, Faculty of Medicine, McGill University. Rue de la Montaigne, Montreal, QC, Canada; 3 Biotechnology Program, University of British Columbia, West Mall, Vancouver, BC, Canada; 4 Experimental Medicine, Faculty of Medicine, McGill University. Rue de la Montaigne, Montreal, QC, Canada; 5 Division of Cellular Immunology, German Cancer Research Center, Heidelberg, Germany; INSERM, FRANCE

## Abstract

In the adult skeleton, cells of the immune system interact with those of the skeleton during all phases of bone repair to influence the outcome. Mast cells are immune cells best known for their pathologic role in allergy, and may be involved in chronic inflammatory and fibrotic disorders. Potential roles for mast cells in tissue homeostasis, vascularization and repair remain enigmatic. Previous studies in combined mast cell- and Kit-deficient *Kit*^*W-sh/W-sh*^ mice *(Kit*^*W-sh*^) implicated mast cells in bone repair but *Kit*^*W-sh*^ mice suffer from additional Kit-dependent hematopoietic and non- hematopoietic deficiencies that could have confounded the outcome. The goal of the current study was to compare bone repair in normal wild type (*WT*) and *Cpa3*^*Cre/+*^ mice, which lack mast cells in the absence of any other hematopoietic or non- hematopoietic deficiencies. Repair of a femoral window defect was characterized using micro CT imaging and histological analyses from the early inflammatory phase, through soft and hard callus formation, and finally the remodeling phase. The data indicate 1) mast cells appear in healing bone of *WT* mice but not *Cpa3*^*Cre/+*^ mice, beginning 14 days after surgery; 2) re-vascularization of repair tissue and deposition of mineralized bone was delayed and dis-organised in *Cpa3*^*Cre/+*^ mice compared with *WT* mice; 3) the defects in *Cpa3*^*Cre/+*^ mice were associated with little change in anabolic activity and biphasic alterations in osteoclast and macrophage activity. The outcome at 56 days postoperative was complete bridging of the defect in most *WT* mice and fibrous mal-union in most *Cpa3*^*Cre/+*^ mice. The results indicate that mast cells promote bone healing, possibly by recruiting vascular endothelial cells during the inflammatory phase and coordinating anabolic and catabolic activity during tissue remodeling. Taken together the data indicate that mast cells have a positive impact on bone repair.

## Introduction

It has been proposed that the discreet phases of bone repair in response to injury recapitulate those during development that give rise to the adult skeleton [1]. It was recognized decades ago that cells of the immune system interact with those of the skeletal system during development and in the adult bone healing micro-environment. The term “osteoimmunology” was coined to define these complex interactions between lymphocytes, macrophages, mast cells, osteoclasts, osteoblasts and others [2]. In the long bones of the adult skeleton the bone healing cascade is initiated with a blood clot and an inflammatory response during which cells migrate to the site of injury [3]. The hematoma is replaced by granulation tissue to form a soft callus, metalloproteases cleave collagen and stored growth factors and cytokines are released. Angiogenic factors attract vascular endothelial cells, which form vessels throughout the repair tissue. Bone anabolic agents such as Wnt ligands, parathyroid hormone (PTH) and related protein (PTHrP) and bone morphogenetic proteins (BMPs) induce differentiation of mesenchymal stromal cells (MSC) into osteoblasts. Woven bone is deposited by these cells in and around the soft callus to form a hard callus, which is then remodeled by osteoclasts delivered through the new vessels.

Mast cells belong to the hematopoietic system and mast cell committed progenitors have been identified in fetal and adult mouse blood [4, 5]. They migrate to peripheral tissues such as lung, skin and intestine where mature mast cells are stored over the long term [6]. Mast cells are best known for their pathologic role in allergic diseases. Less well established are their suggested physiologic roles in tissue homeostasis and repair that include neo-vascularization [7]. Mast cells contain the proteases tryptase and chymase along with a variety of cytokines and chemokines that contribute to allergic inflammation, but some of which may also act as mediators of tissue repair. These include TNFα, prostaglandin (PG) D2, leukotriene (LT) C4, monocyte chemoattractant protein (MCP), macrophage inflammatory protein (MIP) and a plethora of interleukins (ILs). The functional heterogeneity of mast cells is proposed to arise from differential activation of the FcεR1 by IgE or activation of a wide range of pattern recognition toll like receptors (TLR). Generally speaking, FcεR1 activation is proposed to trigger release of proteases and cytokines stored in granules, whereas TLR activation results in de novo synthesis and release of a different sub-set of bioactive mediators [8].

An early study of fracture repair in young rats revealed mast cells adjacent to blood vessels in the soft callus at two weeks of healing and distributed throughout the hard callus near osteoclasts at six weeks [9], suggesting these cells played a role in the healing process. A study conducted under steady state on mast cell-deficient mice carrying a mutation in the receptor for stem cell factor Kit (*Kit* ^*W/Wv*^) revealed alterations in femoral bone mass and geometry leading to decreased mechanical strength [10]. However, the *Kit*^*W/Wv*^ mice also exhibited anemia, neutropenia and other defects [11] that could have impacted the outcome. Our recent work on another strain of Kit mutant mice, *Kit*^*W-sh*^, revealed that bone regeneration in a femoral defect was defective in these mice (Behrends 2014), raising the intriguing possibility that mast cells are involved in bone repair. *Kit*^*W-sh*^ mice were originally suggested to be mast cell deficient in the absence of other major hematopoietic cell deficiencies [12]. However, subsequent work showed that *Kit*^*W-sh*^ mice suffer from a multitude of phenotypes beyond the mast cell deficiency [13–15].

Mouse mutants specifically lacking individual immune cell lineages are powerful resources to investigate the osteoimmunology of bone healing. Until now, mast cell deficient mice wildtype for Kit have not been used to this end. The goal of the current study was to characterize the impact of mast cell deficiency on bone repair using *Cpa3*^*Cre/+*^ mice. Introduction of Cre recombinase into the gene encoding carboxypeptidase A3 (Cpa3), which is expressed at very high levels only in the mast cell lineage, results in the complete absence of mast cells in connective and mucosal tissues by genotoxicity [16]. This model of mast cell deficiency has been used by many investigators to probe roles of mast cells under physiological and pathological conditions. Of note, in almost all instances, the suggested roles of mast cells could not be reproduced, thus contesting previous work in Kit mutant mice [11, 15]. Because *Cpa3*^*Cre/+*^ mice have no defects in the immune system other than the complete absence of mast cells and a partial reduction in basophils, we consider these mice an appropriate model to investigate the potential contribution of mast cells to bone repair in the absence of confounding factors arising from alteration in other cell lineages.

## Materials and methods

### Mouse model of mast cell deficiency

Animal procedures were conducted in accordance with a protocol approved by the Facility Animal Care Committee of McGill University (AUP-7016), in keeping with the guidelines of the Canada Council on Animal Care. Animal surgery and post mortem analyses were performed essentially as described previously [17–19]. Founder mice heterozygous for insertion of *Cre* recombinase in the gene encoding mouse mast cell Cpa3 (*Cpa3*^*Cre/+*^ mice on the *C57BL/6* background) were obtained from the German Cancer Research Center, DKFZ, Heidelberg, Germany. A colony was established by mating with WT *C57BL/6* mice (Charles River Laboratories, Senneville, Qc H9X 3R3, Canada) and the offspring genotyped as described [16] using PCR of DNA isolated from ear punch biopsies. *Cpa3*^*Cre/+*^ mast cell-deficient male and female offspring were separated and 3–4 mice/cage maintained with free access to food and water from 4.5 to 8 months prior to surgical intervention.

### Surgical model

Adult male and female mice were used for all experiments. Bilateral 1 mm x 2 mm defects were generated on the anterolateral aspect of the femora using the third trochanter as an anatomical landmark. Mice were anesthetized with isoflurane before shaving both hind limbs, disinfecting the skin with 70% ethanol and exposing the anterolateral aspect of the femora through a 3-mm skin incision extending from the third trochanter down the diaphysis. A 1 mm x 1 mm x 2 mm rectangular cortical window defect was generated with a 1 mm burr on Stryker drill (50,000 RPM, Hamilton, ON, Canada). After gentle irrigation to remove bone shards the muscle and skin layers were re-apposed and sutured with PDS-II 4–0 thread. For pain control, an IP injection of 10 mg/kg carprofen with 0.1 mg/kg buprenorphine in 0.5 mL of sterile saline was administered immediately after wound closure, and 5 mg/kg carprofen injected for 3 days post-operative. Cohorts of mice were euthanized by CO2 asphyxiation under anesthesia from 5–56 days post-operative. Femora were carefully dissected free of soft tissue before fixing for 24 hours in 4% paraformaldehyde. The bones were then rinsed x3 with sterile PBS and stored at 4o C until micro computed tomographic (micro CT) imaging.

### Micro CT analysis

Scans were performed on a Skyscan 1172 instrument (Bruker, Kontich, Belgium) with a 0.5 mm aluminium filter at a voltage of 50kV, a current of 200μA and a resolution of 5 μm/pixel. 2D images were reconstructed into 3D models using NRecon software v.1.6.10.4 (Bruker) and loaded into CTAn software v.1.16.4.1 (Bruker) for analysis. Quantitative data was recorded for bone and vessel regeneration in rectangular regions of interest (ROI) in the Cortex opposite the defect, measuring 1.5 mm long, 0.9 mm wide and 0.6 mm in depth and in the Defect/Medulla measuring 1.5 mm long, 1.0 mm wide and 1.3 mm in depth. Quantitative data for mineralized tissue includes bone volume/tissue volume (BV/TV %), trabecular thickness (Tb.Th. mm x10-3), trabecular separation (Tb.Sp. mm x10-3), open porosity (%) and open pore volume (Po.V Tot mm3). Quantitative data for vascular channels (Bruker micro CT academy 2016 v5.3) includes vascular channel volume/tissue volume (ChV/TV %), vascular channel number (Ch.N), vascular channel thickness (Ch.Th mm x10-3), and vascular channel connectivity density (Ch.Conn.Dn.).

### Histological analysis

Bones were either left un-decalcified and embedded in poly-methyl methacrylate (PMMA) plastic or decalcified and embedded in paraffin using established methodology. Skin and bone tissues were stained for mast cells using acidified toluidine blue (aTB) and anti- tryptase antibody (Abcam, Cambridge MA, USA ab 151757 1:300). CD34 (Abcam ab23830 1:300) immunohistochemistry was used to identify vascular endothelial cells in bone and soft tissue in regenerating bone. Sections of PMMA embedded bones were stained with von Kossa and counterstained with toluidine blue (VK/TB) to distinguish mineralized from soft tissue. VK/TB stained sections were compared with 2D micro CT images from the same region, or with sections of decalcified, paraffin embedded bone stained to identify alkaline phosphatase (ALP) activity in osteogenic cells. Tartrate resistant acid phosphatase (TRAP) histochemical staining was used to identify osteoclasts and F4/80 (Abcam ab6640 1:200) immunohistochemistry to identify macrophages in decalcified bone sections. Microscopic images were captured with a Zeiss Axioskop 40 microscope (Carl Zeiss, Toronto, ON, Canada) and stain intensity expressed as % of the ROI using ImageJ v.1.6.0 software (NIH, Bethesda, MD, USA).

### Statistical analysis

Quantitative data is expressed as mean ± SD and the open source statistical program R v.3.3.0 (R Core Team, 2015) used for Wilcoxon sum-rank tests. Analysis of variance (ANOVA) followed by Tukey post-hoc analysis were used for longitudinal and multiple comparisons between the different time points in the WT and *Cpa3*^*Cre/+*^mice. Differences were considered significant at p <0.05.

## Results

### Distribution of mast cells during bone repair

**Fig 1:**
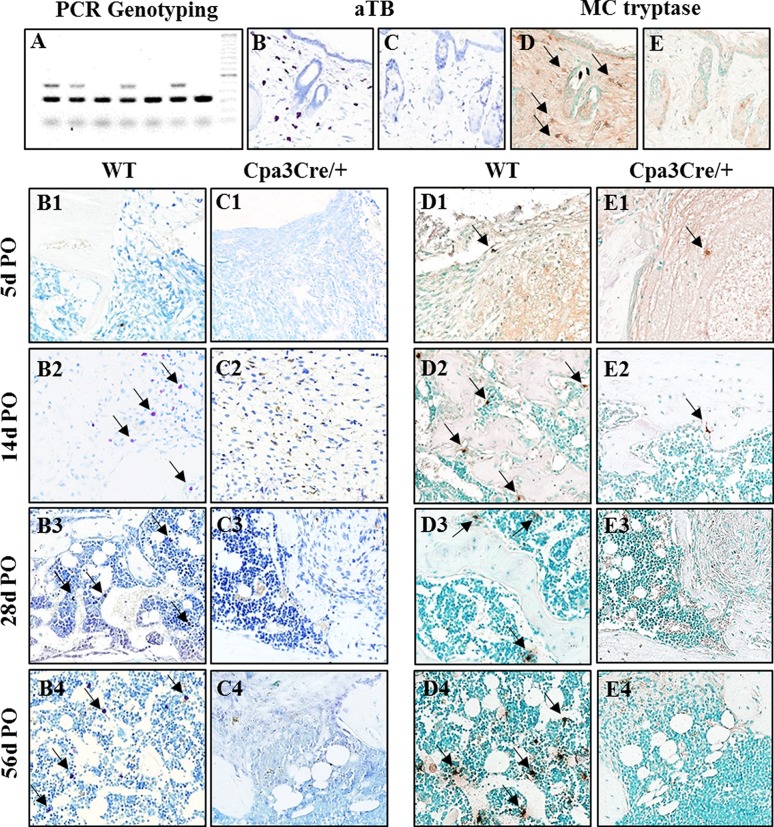
Identification of mature and immature mast cells. Mice heterozygous for knock-in of *Cre* recombinase in the first exon of the *Cpa3* gene were generated by backcross of founder *Cpa3*^*Cre/+*^ mice, shipped from the colony in Heidelberg, Germany, to *C57Bl6* mice. PCR genotyping (A) of DNA isolated from ear punch specimens of 4-week-old mice yields 320bp and 450bp products for heterozygous *Cpa3*^*Cre/+*^ mice. Back skin biopsies were harvested from N = 3 wild type (*WT*, B, D) and N = 3 *Cpa3*^*Cre/+*^ (C, E) mice were embedded in paraffin and adjacent sections stained with acidified toluidine blue (aTB) or immunochemically with mast cell (MC) tryptase to identify mast cells. Representative images show numerous purple stained granular mast cells in *WT* (B) but not in *Cpa3*^*Cre/+*^ (C) skin. MC tryptase immunopositive cells (D arrows) are also seen in *WT* but not in *Cpa3*^*Cre/+*^ skin (E). Bilateral drill hole defects were generated in the femora of adult N = 37 *WT* and N = 35 *Cpa3*^*Cre/+*^ mice and cohorts of animals euthanized from 5d to 56d post-operative (PO). Sections of bone stained with aTB show mature mast cells (arrows) in marrow adjacent to the defect in *WT* (B2-B4) but not in *Cpa3*^*Cre/+*^ (C1-C4) mice. MC tryptase positive cells (arrows) are seen in *WT* tissue starting at 14d PO (D2-D4 but not in *Cpa3*^*Cre/+*^ mice (E2-E4). Images are representative of N = 7 *WT* and N = 6 *Cpa3*^*Cre/+*^ at 5d PO; N = 13 *WT* and N = 10 *Cpa3*^*Cre/+*^ at 14d PO; N = 8 *WT* and N = 10 *Cpa3*^*Cre/+*^ at 28d PO and N = 5 *WT* and N = 8 *Cpa3*^*Cre/+*^ at 56d PO.

Mice were genotyped into *Cpa3*^*+/+*^ (mast cell proficient; *WT*) and *Cpa3*^*Cre/+*^ (mast cell deficient) littermates by PCR (Fig 1, panel A). Mast cells are normally distributed throughout the dermis [20]. Skin biopsies harvested from the backs of adult *WT* and *Cpa3*^*Cre/+*^mice were stained with aTB and for MC tryptase. Numerous aTB (Fig 1, panel B) and MC tryptase positive (Fig 1, panel D) cells were seen in the dermis, usually around hair follicles, in all *WT* (Fig 1, panel A) but not in *Cpa3*^*Cre/+*^ (Fig 1, panels C and E) mice. The same strategy was used to identify mast cells in sections of regenerating bone harvested from mice euthanized from 5d-56d PO. At 5d PO aTB positive cells were occasionally seen in *WT* mice on the periosteal surface of the proximal femur outside the region of the defect. At 14d PO they were seen in residual connective tissue in the defect/medulla of *WT* mice (Fig 1, panel B2) and in marrow, usually adjacent to vascular channels at 28d and 56d PO (Fig 1, panels B3 and B4). aTB positive cells were never seen in any of the bones harvested from *Cpa3*^*Cre/+*^ mice (Fig 1, panels C1 to C4). Quantitative data for mast cell staining, expressed as the number of aTB positive cells/mm^2^, is shown in Table 1. Non-granular, anti-tryptase antibody reactive cells of undefined lineage were seen in fibrous tissue at 5d and 14d PO in the defect/medulla of *WT* (Fig 1, panels D1 and D2) mice and occasionally in *Cpa3*^*Cre/+*^ (Fig 1, panels E1 and E2) mice. At 28d and 56d PO the distribution pattern of MC tryptase positive cells in *WT* mice resembled that of aTB positive cells, adjacent to bone and in the marrow next to vascular channels (Fig 1, panels D3 and D4). Tryptase positive cells were not seen in *Cpa3*^*Cre/+*^ bone at these times (Fig 1, panels E3 and E4).

**Table 1:** Quantitative staining analysis of cellular activity.

	5 days postoperative			14 days postoperative			28 days postoperative			56 days postoperative		
	WT	*Cpa3^Cre/+^*	p value	WT	*Cpa3^Cre/+^*	p value	WT	*Cpa3^Cre/+^*	p value	WT	*Cpa3^Cre/+^*	p value
	(n = 6–10)	(n = 7–9)		(n = 6–13)	(n = 7–10)		(n = 8–9)	(n = 8–10)		(n = 5–6)	(n = 5–9)	
**Cortex**												
aTB (#/mm^2^)	0	0	1.000	0.4 ± 1.1^b^	0	0.268	1.6 ± 1.3^b^	0	**0.001**	0.7 ± 0.7^b^	0	**0.016**
CD34 (%)	0.35±0.20	0.24±0.21	0.309	0.69±0.35^a^	0.70±0.28^b^	0.942	0.80±0.19^b^	1.31±0.26^b^	**0.002**	0.63±0.18	1.02±0.28^b^	**0.046**
ALP (%)	2.22±1.08	2.25±1.98	0.970	5.09±1.34^a^	6.59±2.48^b^	0.090	7.41±2.19^b^	5.98±2.13^a^	0.192	9.08±3.02^b^	7.64±2.98^b^	0.410
TRAP (%)	0.07±0.12	0.01±0.02	0.232	3.70±2.33^b^	3.88±3.80^b^	0.898	1.33±0.61	2.63±1.39^a^	**0.025**	0.57±0.28	1.30±0.62^a^	**0.025**
F4/80 (#/mm^2^)	0.16±0.06	0.14±0.13	0.774	0.28±0.08	0.39±0.13^a^	0.103	0.21±0.06	0.40±0.19^a^	**0.017**	0.44±0.21^a^	0.21±0.09	0.081
**Defect/Medulla**												
aTB (#/mm^2^)	0	0	1.000	10.0 ± 5.9^b^	0	**<0.001**	8.0 ± 3.4^b^	0	**<0.001**	4.9 ± 4.1^b^	0	**0.005**
CD34 (%)	0.31±0.31	0.14±0.10	0.161	0.87±0.40^b^	0.92±0.57^b^	0.810	0.27±0.09	0.45±0.18^a^	**0.035**	0.10±0.06	0.22±0.05	**0.016**
ALP (%)	0.01±0.01	0.02±0.03	0.384	5.16±1.92^b^	4.37±2.11^b^	0.378	3.54±1.25^b^	2.14±0.84^a^	**0.014**	2.09±0.78^a^	2.11±1.54^a^	0.983
TRAP (%)	0.35±0.44	0.01±0.02	**0.047**	5.56±2.99^b^	3.68±2.13^b^	0.122	1.10±0.63	1.63±0.85^a^	0.161	0.29±0.15	0.82±0.30^a^	**0.002**
F4/80 (#/mm^2^)	0.72±0.51	0.36±0.29	0.171	3.59±1.24^b^	2.27±0.67^b^	**0.047**	4.03±0.74^b^	2.96±0.64^b^	**0.009**	3.05±0.49^b^	3.38±0.76^b^	0.486

aTB mast cells; CD34 vascular endothelial cells; ALP osteoblasts; TRAP osteoclasts; F4/80 macrophages. Significantly different from 5 days postoperative

^a^ p <0.05 and

^b^ p <0.01.

### Micro CT analysis of bone repair

**Fig 2:**
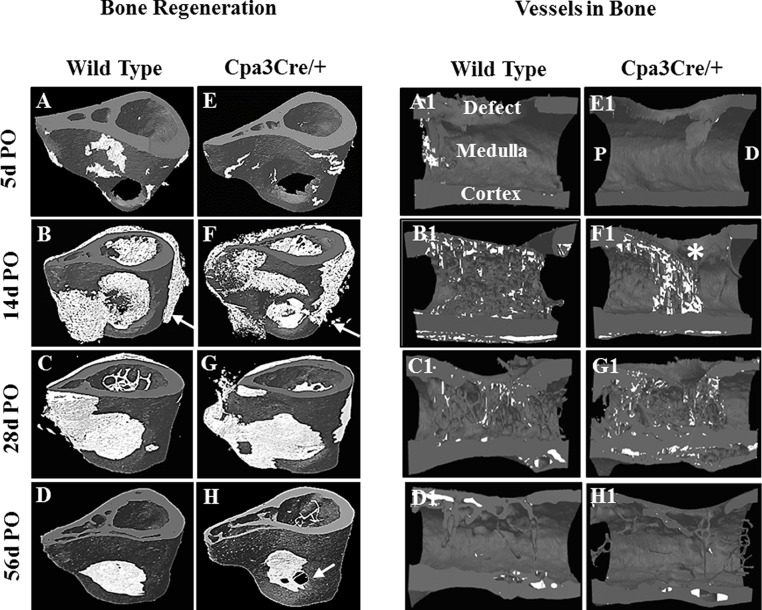
Micro CT analysis of bone and vessel regeneration. Femora harvested from *WT* and *Cpa3*^*Cre/+*^ mice at the indicated time points were scanned at a resolution of 5μm on a Skyscan 1172 instrument. Representative 3D models (A-H), reconstructed from 2D images were tilted at a 45° angle to show healing of the defect over time. At 14d PO (B, F) significant new bone (white) is seen in the medullary canal and on the periosteal surface at the level of the defect (B, F arrows). By 56d PO, bone regeneration and remodelling have effectively closed the defect in the *WT* femur whereas mal-union is evident in the *Cpa3*^*Cre/+*^ femurs, with holes penetrating the new cortical bone (H arrow). 3D models of hemi-femora (A1-H1) show the distribution of blood vessels (white) at the same time points. Revascularization of bone reaches a peak at 14d postoperative, with a skewed distribution in *Cpa3*^*Cre/+*^ mice (F1 asterix), and is restricted to cortical bone by 56d PO (D1, H1).

2D micro CT images were reconstructed into 3D models in which new bone (white) is distinguished from pre-existing bone (dark grey) (Fig 2, panels A to H). A complex healing pattern was seen in the *Cpa3*^*Cre/+*^ mice that differed from that seen in the *WT* mice. At 14d PO less of the periosteal surface opposite the defect was covered with new bone in *WT* (Fig 2, panels B and C) mice than in *Cpa3*^*Cre/+*^ (Fig 2, panels F and G arrows) mice. By 56d PO, bridging of the defect was most often complete in *WT* (Fig 2, panel D) mice but remained incomplete with residual fibrous tissue in most *Cpa3*^*Cre/+*^ mice (Fig 2, panel H arrow). Quantitative analysis of bone regeneration in the cortex and defect/medulla ROIs is shown in Table 2. By 14d PO the *WT* mice had less cortical bone with wider spaces between trabeculae (Tb.Sp.) and increased bone porosity (Po.Vop; Po.op) compared with *Cpa3*^*Cre/+*^ mice, but with little difference in the defect/medulla. By 56d PO BV/TV was higher, with narrower spaces and less porosity, in WT than in *Cpa3*^*Cre/+*^ mice in both cortex and defect/medulla.

**Table 2:** Quantitative micro CT analysis of bone architecture.

	5 days postoperative			14 days postoperative			28 days postoperative			56 days postoperative		
	WT	*Cpa3^Cre/+^*	p value	WT	*Cpa3^Cre/+^*	p value	WT	*Cpa3^Cre/+^*	p value	WT	*Cpa3^Cre/+^*	p value
	(n = 7)	(n = 6)		(n = 16)	(n = 10)		(n = 8)	(n = 10)		(n = 6)	(n = 8)	
**Cortex**												
BV/TV %	33.6±4.7	30.8±3.3	0.112	34.4±4.1	41.7±4.6^b^	**<0.001**	39.0±3.8^b^	41.1±7.6^b^	0.370	42.0±4.5^b^	36.3±6.5	**0.025**
Tb.Th. (mm x10^-3^)	20.7±2.0	19.2±1.2	**0.031**	17.7±2.4^b^	16.8±2.0^a^	0.242	17.1±2.7^b^	16.00±2.6^b^	**0.001**	18.0±1.9^a^	16.4±2.5	0.133
Tb. Sp (mm x10^-3^)	29.1±3.1	30.4±2.8	0.304	29.1±5.4	19.5±5.2^b^	**0.001**	29.5±2.19	23.4±5.0^b^	**0.003**	24.3±3.3^b^	28.3±3.4	**0.021**
Po.Vop (mm^3^)	0.54±0.04	0.56±0.03	0.117	0.54±0.05	0.47±0.06^b^	**0.001**	0.49±0.04^a^	0.48±0.06^b^	0.601	0.47±0.04^b^	0.56±0.06	**0.002**
Po.op %	66.4±4.7	69.2±3.3	0.113	65.6±4.1	58.3±4.6	**0.001**	60.9±3.8b	58.9±7.4	0.373	58.0±4.5^b^	63.7±6.5	**0.025**
**Defect/Medulla**												
BV/TV %	0.95±0.73	0.53±0.69	0.163	9.23±6.16^b^	8.44±5.39^b^	0.660	22.9±8.4^b^	16.3±6.5^b^	**0.018**	22.6±4.5^b^	16.8±7.09^b^	**0.032**
Tb.Th. (mm x10^-3^)	4.67±0.02	3.73±1.51	0.178	3.24±0.64^b^	3.38±0.52	0.456	5.81±0.86^b^	5.28±0.86^b^	0.165	9.31±0.74^b^	8.0±2.1^b^	0.088
Tb. Sp (mmx10^-3^)	59.7±3.1	61.7±1.8	0.075	28.5±10.3^b^	29.9±14.4^b^	0.707	20.5±5.6^b^	21.7±4.5^b^	0.584	25.1±4.0^b^	30.7±4.6^b^	**0.016**
Po.Vop (mm^3^)	78.4±3.1	77.8±0.8	0.575	71.1±5.2^b^	71.2±4.5	0.954	60.3±5.9^b^	65.4±5.1	**0.032**	60.8±3.6^b^	67.7±8.4^a^	**0.034**
Po.(op) %	99.0±0.75	99.4±0.83	0.239	90.9±6.0^b^	91.6±5.5^b^	0.683	76.3±8.6^b^	82.5±6.9^b^	**0.031**	75.3±5.8^b^	83.3±7.5^b^	**0.010**

BV/TV = bone volume/tissue volume; TbTh = trabecular thickness; TbSp. = trabecular separation; PoVop = open pore volume; Po(op) = open porosity. Significantly different from 5 days postoperative

^a^ p <0.05 and

^b^ p <0.01

A new algorithm developed by Bruker was used to quantify vessels (Fig 2, panels A1 to H1) in bone (Bruker micro CT academy 2016 v5.3). At 5d PO vessels were starting to penetrate the repair tissue from the proximal femur in *WT* mice but were not yet visible in *Cpa3*^*Cre/+*^ mice. By 14d PO there was a dramatic increase in vessels in both *WT* and *Cpa3*^*Cre/+*^ bones. Whereas the distribution pattern was even in *WT* mice it was skewed to the proximal pole leaving a distal area with no penetration (asterix) and few cortical vessels in *Cpa3*^*Cre/+*^ mice. By 56d PO the new vessels were effectively restricted to cortical bone. Quantitative analyses for new vessels is shown in Table 3. At 5d PO there were more vessels with a higher volume and better connectivity in *WT* than in *Cpa3*^*Cre/+*^ bone, and the number, volume and thickness remained higher at 56d PO. Images are representative of N = 7 *WT* and N = 6 *Cpa3*^*Cre/+*^ at 5d PO; N = 16 *WT* and N = 11 *Cpa3*^*Cre/+*^ at 14d PO; N = 8 *WT* and N = 10 *Cpa3*^*Cre/+*^ at 28d PO and N = 6 *WT* and N = 8 *Cpa3*^*Cre/+*^ at 56d PO.

**Table 3:** Quantitative micro CT analysis of bone vasculature.

	5 days postoperative			14 days postoperative			28 days postoperative			56 days postoperative		
	WT	*Cpa3^Cre/+^*	p value	WT	*Cpa3^Cre/+^*	p value	WT	*Cpa3^Cre/+^*	p value	WT	*Cpa3^Cre/+^*	p value
	(n = 7)	(n = 6)		(n = 16)	(n = 10)		(n = 8)	(n = 10)		(n = 6)	(n = 8)	
**Cortex**												
Ch.V/TV %	0.43±0.4	0.26±0.2	0.264	9.8±5.8^b^	11.2±4.8^b^	0.482	2.04±1.7	4.5±2.4^b^	**0.003**	1.73±0.8	1.83±1.1	0.814
Ch.N.	0.33±0.3	0.11±0.09	0.103	2.6±1.3^b^	3.21.6^b^	0.201	0.61±0.4	1.04±0.4^a^	**0.021**	0.37±0.1	0.43±0.2	0.331
Ch.Th. (mm x10^-3^)	16.8±7.4	19.7±12.0	0.537	32.6±9.4^b^	36.1±9.0^b^	0.224	30.8±8.0^b^	41.0±12.8^b^	**0.009**	46.1±14.7^b^	40.8±15.4^b^	0.471
Ch.Conn.Dn.	0.15±0.2	0.008±0.005	**0.031**	1.18±0.8^b^	1.48±1.3^b^	0.403	0.07±0.07	0.20±0.18	**0.001**	0.06±0.05	0.06±0.03	0.923
**Defect/Medulla**												
Ch.V/TV %	9.04±8.7	3.5±3.1	**0.039**	38.61±7.2^b^	28.71±13.4^b^	**0.006**	10.74±4.7	12.12±3.7^a^	0.401	3.58±2.25	1.44±0.8	**0.022**
Ch.N.	4.1±3.0	2.0±1.5	**0.036**	13.1±2.5^b^	10.1±4.1^b^	**0.008**	3.9±2.0	4.5±1.6^a^	0.548	1.0±0.3^a^	0.9±0.3	0.431
Ch.Th. (mm x10^-3^)	19.3±6.9	18.0±3.2	0.667	31.1±2.8^b^	25.9±7.4^b^	**0.007**	28.6±8.8^a^	27.9±5.9^b^	0.801	35.6±16.2^b^	23.1±5.8	**0.039**
Ch.Conn.Dn.	1.69±1.5	0.5±0.4	**0.031**	8.62±3.6^b^	6.1±3.1^b^	**0.019**	1.19±1.1	1.5±0.9	0.452	0.33±0.2	0.21±0.1	0.115

Ch.V/TV % = channel volume/tissue volume; Ch.N. = channel number; Ch.Th. (mm x10^-3^) = channel thickness; Ch.Conn.Dn. = channel connectivity density. Significantly different from 5 days postoperative

^a^ p <0.05 and

^b^ p <0.01

**Fig 3:**
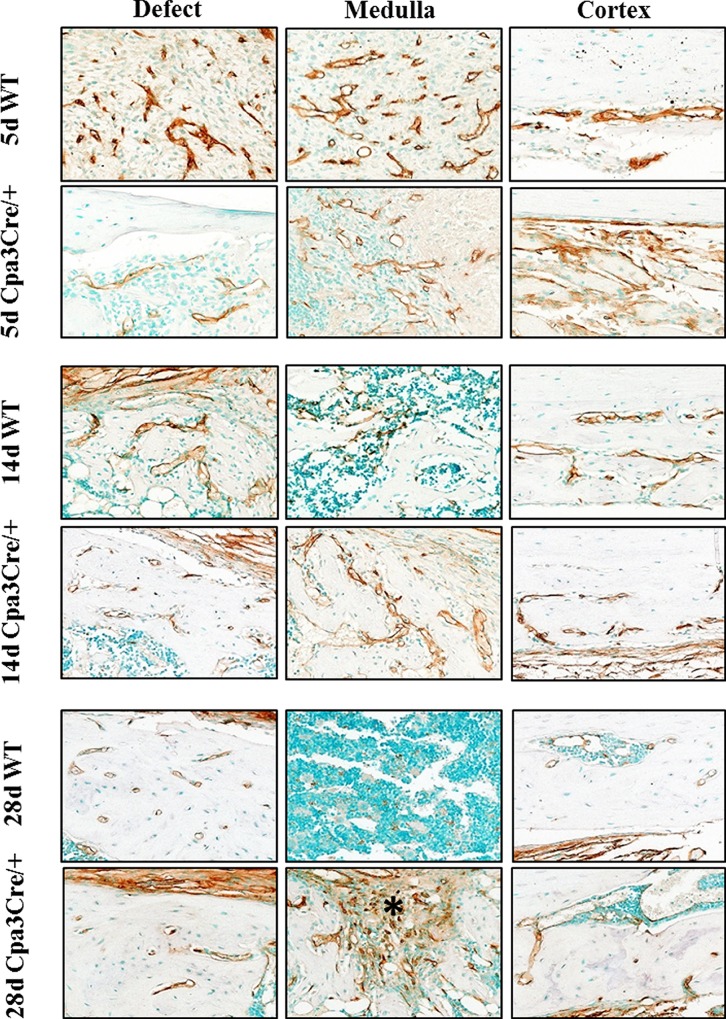
CD34 immunohistochemistry in regenerating bone. Bones were decalcified, embedded in paraffin and 5μm sections stained immunochemically for CD34 expression. Representative images of the defect, medulla and contralateral cortex show robust staining of cells lining vessels at 5d PO in *WT* bone compared with weak, disorganised staining in *Cpa3*^*Cre/+*^ bone. By 28 days PO CD34 immunoreactivity is restricted primarily to the periosteum in *WT* bone, but persists in the remaining fibrous tissue in *Cpa3*^*Cre/+*^ bone (asterix). Images are representative of N = 7 *WT* and N = 6 *Cpa3*^*Cre/+*^ at 5d PO; N = 11 *WT* and N = 9 *Cpa3*^*Cre/+*^ at 14d PO and N = 8 *WT* and N = 6 *Cpa3*^*Cre/+*^ at 28d PO.

The localization of vessels in regenerating bone was then compared with the results of CD34 immunohistochemistry, which was used as a sensitive marker for vascular endothelial cells in soft tissue and bone (Fig 3). At 5d and 14d PO there was an extensive network of vessels in the defect, medulla and cortex of *WT* bones, compared with the less dense pattern of vessel distribution seen in *Cpa3*^*Cre/+*^ bones. By 28d PO there were few CD34 positive cells in the medulla of *WT* mice compared with numerous cells embedded in fibrous tissue remaining in the defect and medulla of *Cpa3*^*Cre/+*^ bones (asterix). Quantification of CD34 staining, shown in Table 1, revealed peak activity at 14d PO in the defect/medulla and at 28d PO in the cortex of both *WT* and *Cpa3*^*Cre/+*^ bones, but with fewer vessels at 28d and 56d PO in the *WT* mice.

### Histological analysis of bone repair

**Fig 4:**
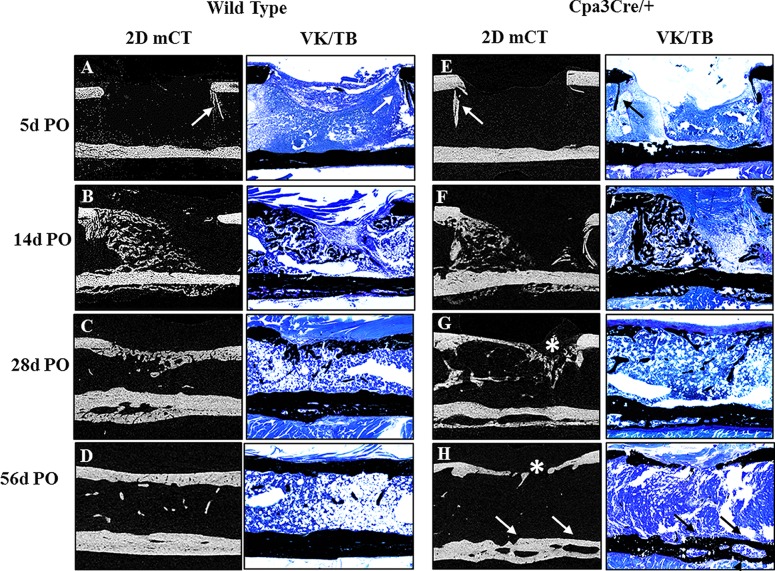
Macroscopic evaluation of bone repair over time. 2D micro CT images (A-H) were compared with 5 μm histological sections of un-decalcified bone stained with von Kossa and toluidine blue (VK/TB) to distinguish mineralised (black) from un-mineralized tissue (blue). Representative mid-sagittal images show shards of old bone (A, E arrows) remaining in the defect at 5d PO and significant new bone at 14d PO in the medullary canal and on the periosteal surface opposite the defect in both *WT* (B) and *Cpa3*^*Cre/+*^ (F) femora. At 28 days PO, the defect is bridged with primary bone in many *WT* (C), but not *Cpa3*^*Cre/+*^ (G asterix) mice. By 56d PO the majority of *WT* femora have assumed their pre-operative anatomy (D), whereas most of those from *Cpa3*^*Cre/+*^ mice exhibit mal-union on the defect side (H asterix) and large channels separating old from new bone on the contralateral cortex (H arrows). Images are representative of N = 7 *WT* and N = 6 *Cpa3*^*Cre/+*^ at 5d PO; N = 16 *WT* and N = 11 *Cpa3*^*Cre/+*^ at 14d PO; N = 8 *WT* and N = 10 *Cpa3*^*Cre/+*^ at 28d PO and N = 6 *WT* and N = 8 *Cpa3*^*Cre/+*^ at 56d PO.

To further characterize the quality of regenerated bone we stained thin sections of un-decalcified bone harvested from the mid-saggital plane of the defect with von Kossa (Fig 4) to compare with 2D micro CT images. The morphological features seen in the 2D micro CT images (Fig 4, panels A to H) were reflected in the VK/TB stained histological sections. Residual shards of old bone (Fig 4, panels A and E arrows) were seen at 5d PO but no new bone was visible until 14d PO (Fig 4, panels B and F). Bridging of the defect was evident at 28d PO in *WT* but not in *Cpa3*^*Cre/+*^ mice (Fig 4, panel C vs G) in which significant fibrous tissue remained (Fig 4, panel G asterix). By 56D PO bone repair was effectively complete in most *WT* bones (Fig 4, panel D) compared with *Cpa3*^*Cre/+*^ bones, where the defect was filled with thin bone interspersed with fibrous tissue (Fig 4, panel H asterix). The spaces between old and new bone seen in the cortex at 28D were retained only in *Cpa3*^*Cre/+*^ bones (Fig 4, panel H arrows).

**Fig 5:**
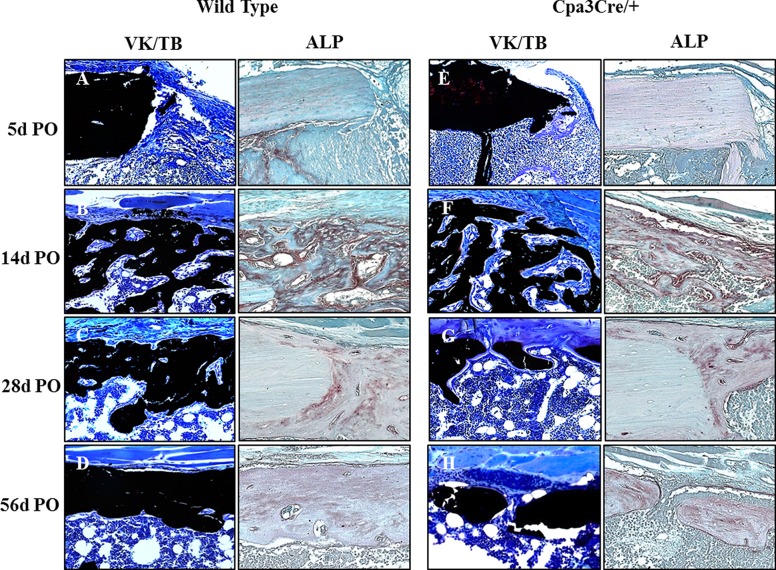
Histochemical analysis of bone in defect/medulla. Representative images of 5 μm sections of un-decalcified bone stained with von Kossa and toluidine blue (A-H) were compared with the equivalent region of 5μm sections of decalcified bone stained with alkaline phosphatase (ALP). Prominent osteoblasts against osteoid seams can be seen at 14d (F) and 28d (G) PO in *Cpa3*^*Cre/+*^ bones compared with *WT* bones (B-C). ALP activity peaks at 14 days PO in both *WT* (B) and *Cpa3*^*Cre/+*^ (F) bones and declines thereafter. Images are representative of N = 7 *WT* and N = 6 *Cpa3*^*Cre/+*^ at 5d PO; N = 16 *WT* and N = 11 *Cpa3*^*Cre/+*^ at 14d PO; N = 8 *WT* and N = 10 *Cpa3*^*Cre/+*^ at 28d PO and N = 6 *WT* and N = 8 *Cpa3*^*Cre/+*^ at 56d PO.

**Fig 6:**
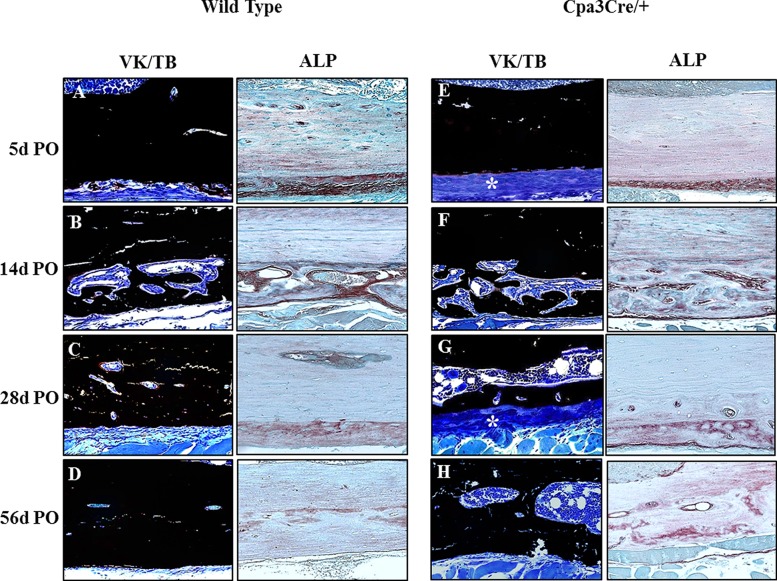
Histochemical analysis of bone in contralateral cortex. Representative images of 5 μm sections of von Kossa stained un-decalcified bone (A-H) were compared with 5μm sections of decalcified bone stained with ALP. A thick, fibrous periosteum is apparent in *Cpa3*^*Cre/+*^ bones (E, G asterix) in the absence of any significant difference in ALP activity. Bone formation with large osteoblasts adjacent to osteoid is apparent at 14d PO in *WT* (B) and *Cpa3*^*Cre/+*^ (F) bones, accompanied by high ALP activity. Active bone formation is sustained at 28d (G) and 56d (H) PO in *Cpa3*^*Cre/+*^ mice, but is less apparent in *WT* mice (C, D). Images are representative of N = 7 *WT* and N = 6 *Cpa3*^*Cre/+*^ at 5d PO; N = 16 *WT* and N = 11 *Cpa3*^*Cre/+*^ at 14d PO; N = 8 *WT* and N = 10 *Cpa3*^*Cre/+*^ at 28d PO and N = 6 *WT* and N = 8 *Cpa3*^*Cre/+*^ at 56d PO.

The cortical window defect is stable and therefore heals through intra-membranous bone formation, with no cartilage intermediate, so ALP is expressed only by anabolic cells of the osteoblast lineage. High magnification images of VK/TB and ALP stained sections from the corresponding region of the defect/medulla (Fig 5) revealed little bone forming activity at 5d PO in either *WT* (Fig 5A) or *Cpa3*^*Cre/+*^ (Fig 5, panel E) bones. At 14d PO there was an extensive network of new bone trabeculae at the proximal end of the defect and in the adjacent medulla, associated with intense ALP activity in both *WT* and *Cpa3*^*Cre/+*^ bone (Fig 5, panels B and F). At 28d and 56d PO there was more bone and less osteoid (blue) spanning the defect in *WT* (Fig 5, panel C) than in *Cpa3*^*Cre/+*^ (Fig 5, panel G) mice, while ALP activity appeared similar. Detailed examination of VK/TB staining in the cortex (Fig 6) revealed less pronounced fibrous periosteal tissue in *WT* (Fig 6, panel A) than *Cpa3*^*Cre/+*^ bone (Fig 6, panel E asterix) at 5d PO, prior to active bone formation at 14d PO when *WT* (Fig 6, panel B) and *Cpa3*^*Cre/+*^ (Fig 6, panel B and F) bones looked similar. At 28d and 56d PO the cortex of *WT* bones (Fig 6, panels C and D) had fewer lacunae and less osteoid than of *Cpa3*^*Cre/+*^ bones (Fig 6, panels G and 6).

**Fig 7:**
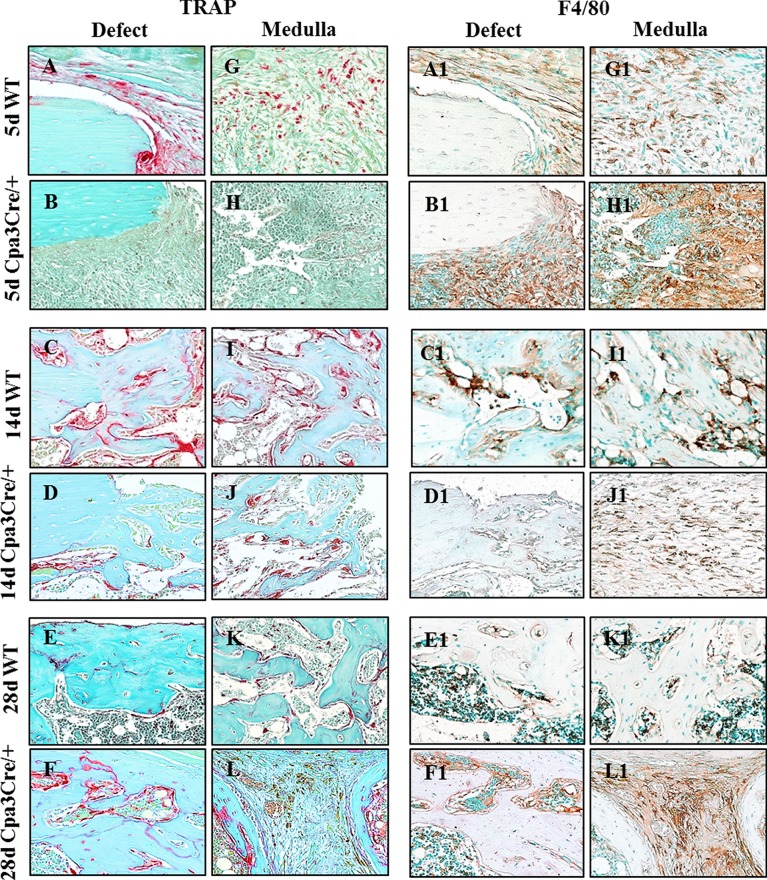
Identification of osteoclasts and macrophages in regenerating bone. 5 μm sections of decalcified bone were stained with tartrate resistant acid phosphatase (TRAP) or immunochemically with the macrophage marker F4/80. Representative images show more TRAP activity in *WT* than in *Cpa3*^*Cre/+*^ bones at 5d (A vs B) and 14d (C vs D) PO, and less at 28d (E vs F) PO. F4/80 positive macrophages were seen in condensed mesenchyme filling the defect/medulla at 5d PO in both *WT* (A1) and *Cpa3*^*Cre/+*^ (B1) bones. In *WT* bone, F4/80 positive cells can be seen lining vessels at 14d (C1) PO and scattered throughout bone marrow at 28d (E1) PO, whereas they were embedded in fibrous tissue in *Cpa3*^*Cre/+*^ bone (F1). Images are representative of N = 7 *WT* and N = 6 *Cpa3*^*Cre/+*^ at 5d PO; N = 16 *WT* and N = 11 *Cpa3*^*Cre/+*^ at 14d PO; N = 8 *WT* and N = 10 *Cpa3*^*Cre/+*^ at 28d PO and N = 6 *WT* and N = 8 *Cpa3*^*Cre/+*^ at 56d PO.

Adjacent sections of decalcified bone were stained with TRAP to identify osteoclasts or with F4/80 antiserum to identify macrophages (Fig 7). TRAP positive cells were seen at the proximal end of the defect and in the fibrous tissue filling the defect at 5d PO in *WT* bones (Fig 7, panel A) but not in *Cpa3*^*Cre/+*^ bones (Fig 7, panel B). Peak TRAP activity occurred at 14d PO and was more intense in *WT* (Fig 7, panel C) than in *Cpa3*^*Cre/+*^ bones (Fig 7, panel D), whereas it declined at 28d PO in *WT* (Fig 7, panel E) but not in *Cpa3*^*Cre/+*^ bones (Fig 7, panel F). F4/80 staining was prominent in connective tissue of *WT* (Fig 7, panel A1) and *Cpa3*^*Cre/+*^ (Fig 7, panel B1) bones at 5d PO. By 14d PO prominent staining was seen adjacent to bone in *WT* (Fig 7, panel C1) but not in *Cpa3*^*Cre/+*^ (Fig 7, panel D1) bones, where F4/80 positive cells remained scattered in connective tissue. By 28d PO the F4/80 positive cells were seen in marrow adjacent to bone in *WT* mice (Fig 7, panel E1) and persisted in fibrous tissue in *Cpa3*^*Cre/+*^ bone (Fig 7, panel F1). Quantitative analyses for ALP, TRAP and F4/80 staining (Table 1) confirmed similar patterns for ALP in *WT* and *Cpa3*^*Cre/+*^ bones throughout the healing period, as well as higher TRAP activity in *WT* bone at 5d PO followed by lower levels than in *Cpa3*^*Cre/+*^ bones at later time points. F4/80 immunoreactivity was generally similar except for transient reductions in *Cpa3*^*Cre/+*^ bones at 14d and 28d PO.

## Discussion

The goal of the current study was to characterize the impact of mast cell deficiency on the repair of cortical bone defects using adult mast cell-deficient *Cpa3*^*Cre/+*^ mice. The *Cpa3*^*Cre/+*^ strain is constitutively devoid of mast cells in connective and mucosal tissues and it has no known alterations in other cell lineages involved in bone repair. In *WT* but not in *Cpa3*^*Cre/+*^ mice mast cells appeared in the repair tissue from 14d to 56d PO. Interestingly, bridging of the bone defect was complete in all (6/6) *WT* mice at 56d PO but only 3/8 *Cpa3*^*Cre/+*^ mice. This incomplete bridging was associated with disruption of re-vascularization and impaired bone mineralization. Osteoclast activity was reduced in *Cpa3*^*Cre/+*^ mice in the early phase of repair but increased at later stages, with no clear differences in macrophage activity. Taken together, the results indicate that mast cells have a positive impact on bone repair that is mediated in part by recruitment of vascular endothelial cells, as also suggested by the previous work of Boesiger *et al* (1998) [21], and in part by altered metabolism of newly formed bone. It was proposed more than two decades ago that mast cells are involved in tissue digestion and re-vascularization, which are early and essential steps in the bone healing cascade [9]. Mast cells reside over the long term in connective tissues where they are available locally and for potential trafficking via the vascular and lymphatic systems to sites of tissue injury and repair [22]. The surgical intervention used in this study would have temporarily disrupted existing hind limb vessels and caused local ischemia and hypoxia, which are the major stimuli for re-vascularization [23]. Mast cells, identified by the use of metachromatic staining with aTB, were first seen in *WT* bone at 5d PO, which was the earliest time at which the soft callus could be preserved intact for histological analyses. At this time they were localized adjacent to vessels in muscle, in bone marrow of the femur proximal to the site of injury, and at the periosteal junction between soft tissue and bone. The appearance of aTB positive cells at the proximal, rather than distal, end of the femur suggests they migrated to the wound from soft tissue stores via the arterial or lymphatic vessels that supply the hind limb. This conjecture was supported by micro CT analyses of vessels in regenerating bone showing initiation of re-vascularization at 5d PO at the proximal end of the defect in *WT* bones. Quantitative analyses revealed peak numbers of aTB positive mast cells in the defect/medulla of *WT* bones at 14d PO, and in the cortex at 28d PO. This timeframe was supported by qualitative data showing MC tryptase positive cells in bone marrow starting at 14d in *WT* but not *Cpa3*^*Cre/+*^ mice. Peak numbers of mast cells in regenerating bone coincided with peak numbers of vessels visualized by micro CT and with CD34 immunostaining of vascular endothelial cells.

Degranulation or secretion of factors by aTB positive mast cells would result in local release of angiogenic mediators like heparin, angiogenin, vascular endothelial growth factor and matrix metalloproteinases in *WT* mice [24]. Given their lack of mast cells these mediators would be absent in *Cpa3*^*Cre/+*^ mice, which could have accounted for the delay and disorganization of bone re-vascularization as evidenced by micro CT. In this and other studies of bone repair we have used CD34 as a sensitive marker of endothelial cells, but it is also expressed on MSC, fibrocytes and other precursor cells capable of differentiating down the osteogenic lineage [25]. In the current work CD34 cells were clearly organized into vascular channels in the condensed mesenchyme in defect/medulla and the cortex at 5d PO prior to bone formation. Consistent with delayed healing, CD34 cells persisted in the *Cpa3*^*Cre/+*^ mice in the periosteum and in residual fibrous tissue in the defect/medulla at 56d PO, when re-vascularization was effectively complete in *WT* mice. The absence of clearly defined channels and the location of CD34 positive cells at 56d PO suggest the cells were not endothelial cells but rather fibrocytes in the periosteum, or MSC that were unable to differentiate into bone-forming osteoblasts in the absence of mast cell mediators. The current literature identifies bone active agents like platelet derived growth factor, fibroblast growth factors, transforming growth factor and tumor necrosis factor, as well as matrix metalloproteinases as pre-formed components of stored granules that are released upon mast cell activation [22, 26]. After 14d PO mast cells were present in close proximity to vessel in the defect/medulla of *WT* mice but absent from *Cpa3*^*Cre/+*^ mice. The absence of mast cell derived bone active agents could have impaired mineralization of newly formed bone at 14d and 28d PO despite similar levels of alkaline phosphatase activity in osteoblasts (Figs 6 and 7). Furthermore, the absence of carboxypeptidase A, which is a major constituent of mast cell granules that targets the vasoconstrictor endothelin [27, 28] could have resulted in excess endothelin-1 mediated vasoconstriction, increased oxidative stress and altered bone cell function [28].

The importance of an adequate periosteal reaction to bone healing is emphasized by the current widespread use of vascularised fibular periosteal grafts to promote healing of large bone defects [29, 30] and in cell-based bone tissue engineering [31]. Prior to skeletal maturity the periosteum is thick and highly vascular, with active osteoblasts depositing intramembranous bone to increase the external diameter of growing long bones [32]. The absence of mast cells in *Cpa3*^*Cre/+*^ mice resulted in thickening of the periosteum, similar to that seen in adult *FGFR3-null* mice [33], and suggests an imbalance in FGF signaling might be involved in impaired bone regeneration in the *Cpa3*^*Cre/+*^ mice. However, the similarity in ALP activity between *WT* and *Cpa3*^*Cre/+*^ mice supports the hypothesis that the impairment was not mediated at an early stage of osteoblast differentiation.

The two principle cells responsible for catabolic activity in bone are osteoclasts and macrophages. Apart from being phagocytes the lineages appear to share little in common. Osteoclast activity is restricted to digestion of mineralized tissue in cartilage and bone whereas macrophages have been implicated in inflammatory disorders and fibrosis of most if not all major organs, including the brain. In our previous work that characterized bone repair in *Kit*^*W-sh*^ mast cell deficient mice osteoclast activity was shown to be elevated throughout the bone healing cascade [18]. This was not the case in the current study in which osteoclast activity was reduced in *Cpa3*^*Cre/+*^ mice up to 14d PO and elevated thereafter compared with *WT* mice. A simple explanation for the discrepancy is that Kit is expressed on osteoclasts and their precursors, as well as mast cells, whereas Cpa3 expression is restricted to mast cells. Osteoclasts are considered to be tissue specific macrophages, distinct from bone marrow macrophages and “osteomacs” located on the periosteal and endosteal surface of resting bone [34]. Using F4/80 immunostaining to identify all macrophages in healing bone it was surprising to find the lowest levels of expression during the inflammatory phase of repair at 5d PO, with an approximate 5-fold increase thereafter in both *WT* and *Cpa3*^*Cre/+*^ mice. The relatively low abundance of macrophages under healing conditions in the absence of mast cells in *Cpa3*^*Cre/+*^ mice in the current study, and previously in *Kit*^*W-sh*^ mice, suggests an interdependence between the two lineages. This conjecture is further supported by the increase in both mast cells and macrophages in bone defects in *WT* mice administered lipopolysaccharide (LPS) [35]. The timeframe and pattern of distribution of F4/80 positive cells in the current study closely resembled that of “osteomacs” in a similar model of cortical bone repair [36]. Given the proposed anabolic function of these cells in bone mineralization [37], their relative short supply in *Cpa3*^*Cre/+*^ bone might explain the increase in osteoid compared with *WT* bone at 14d and 28d PO. Taken together the data suggest a functional relationship exists between mast cells, macrophages and MSC in the bone micro-environment that warrants further investigation. Only few suggested mast cell functions have been confirmed in studies using Kit-independent models of mast cell deficiency. Thus, the impairment of bone fracture healing in mice in the absence of mast cells appears to be a remarkable, largely unanticipated, non-immunological mast cell function.

## Supporting information

S1 File Bone repair in mast cell-deficient mice.xlsx. Dataset to the article.
(XLSX)
